# Patient-specific computer-based decision support in primary healthcare—a randomized trial

**DOI:** 10.1186/1748-5908-9-15

**Published:** 2014-01-20

**Authors:** Tiina Kortteisto, Jani Raitanen, Jorma Komulainen, Ilkka Kunnamo, Marjukka Mäkelä, Pekka Rissanen, Minna Kaila

**Affiliations:** 1 School of Health Sciences, University of Tampere, Tampere, Finland; 2 Pirkanmaa Hospital District, Tampere University Hospital, Tampere, Finland; 3 UKK Institute for Health Promotion Research, Tampere, Finland; 4 Finnish Medical Society Duodecim, Helsinki, Finland; 5 Duodecim Medical Publications Ltd, Helsinki, Finland; 6 Finnish Office for Health Technology Assessment at National Institute for Health and Welfare, Helsinki, Finland; 7 Department of General Practice, University of Copenhagen, Copenhagen, Denmark; 8 The Hjelt Institute, Faculty of Medicine, University of Helsinki, Helsinki, Finland

**Keywords:** Computer-based decision support, Electronic health records, Generalized estimation equation, Primary healthcare, Randomized controlled trial, Reminder systems

## Abstract

**Background:**

Computer-based decision support systems are a promising method for incorporating research evidence into clinical practice. However, evidence is still scant on how such information technology solutions work in primary healthcare when support is provided across many health problems. In Finland, we designed a trial where a set of evidence-based, patient-specific reminders was introduced into the local Electronic Patient Record (EPR) system. The aim was to measure the effects of such reminders on patient care. The hypothesis was that the total number of triggered reminders would decrease in the intervention group compared with the control group, indicating an improvement in patient care.

**Methods:**

From July 2009 to October 2010 all the patients of one health center were randomized to an intervention or a control group. The intervention consisted of patient-specific reminders concerning 59 different health conditions triggered when the healthcare professional (HCP) opened and used the EPR. In the intervention group, the triggered reminders were shown to the HCP; in the control group, the triggered reminders were not shown. The primary outcome measure was the change in the number of reminders triggered over 12 months. We developed a unique data gathering method, the Repeated Study Virtual Health Check (RSVHC), and used Generalized Estimation Equations (GEE) for analysing the incidence rate ratio, which is a measure of the relative difference in percentage change in the numbers of reminders triggered in the intervention group and the control group.

**Results:**

In total, 13,588 participants were randomized and included. Contrary to our expectation, the total number of reminders triggered increased in both the intervention and the control groups. The primary outcome measure did not show a significant difference between the groups. However, with the inclusion of patients followed up over only six months, the total number of reminders increased significantly less in the intervention group than in the control group when the confounding factors (age, gender, number of diagnoses and medications) were controlled for.

**Conclusions:**

Computerized, tailored reminders in primary care did not decrease during the 12 months of follow-up time after the introduction of a patient-specific decision support system.

**Trial registration:**

ClinicalTrial.gov NCT00915304

## Background

The treatment of patients is based on clinical expertise, whose key elements are research evidence, clinical situations and circumstances, and patients’ preferences and actions [1]. The evidence is translated into practical form, for example, in clinical practice guidelines [2], whereas active incorporation of these into everyday practice has only recently become a recognized target for research [3,4]. These methods have previously been summarized and the conclusion is that because there are no magic formulae [5,6], tailoring the intervention is necessary [7].

One of the innovations in the incorporation of evidence into practice is computer-based decision support to bring relevant evidence to the attention of healthcare professionals (HCPs) at the point of care [8]. Such automatic systems combine medical evidence with patient-specific data from the Electronic Patient Record (EPR), which supports clinical decision making [9-11]. According to a Cochrane Review of 28 studies, computer reminders achieved a median improvement in process adherence of 4.2% [12]. Focused computer-generated reminders and alerts work well in a variety of single conditions [13-16] and in preventive care [17]. Decision support can in many settings improve the quality of care and help to avoid mistakes in clinical work, thereby improving patient safety [18,19]. There is still, however, scant evidence on how such information technology solutions work across many diseases or conditions in primary healthcare where multi-professional teams [20,21] care for patients with multiple health problems, both acute and chronic [22,23].

In our study, a set of evidence-based patient-specific reminders in the form of the computer-based decision support service EBMeDS (Evidence-Based Medicine electronic Decision Support, http://www.ebmeds.org) was integrated into the EPR system of one primary care organisation. The EBMeDS service aims to aid treatment across several conditions in actual clinical practice and should therefore be usable in primary healthcare. Our study question was: ‘Do patient- and problem-specific automatic reminders shown to HCPs during primary care consultations have an effect on patient care?’ We hypothesized that the total number of triggered reminders would decrease in the intervention group, in contrast to the control group, indicating a possible improvement in patient care. The hypothesis was formulated on the basis of the idea of a gold standard, by which a triggered reminder indicates that the patient care is not evidence-based, and no reminder indicates that the patient care is evidence-based.

The study was reviewed and accepted by the Pirkanmaa Hospital District (Tampere University Hospital) Ethics Committee (ETL R08149) and registered at ClinicalTrials.gov (www.clinicaltrials.gov: NCT00915304).

## Methods

### Trial design

The setting was the primary healthcare center of Sipoo, which was selected from regular users of the Mediatri EPR system. The center comprises 48 HCPs: 15 physicians, 24 nurses and 9 other HCPs (physiotherapists, ward nurses, a psychologist), described in detail elsewhere [24]. The HCPs used the EPR system during outpatient consultations as well as on an inpatient ward typical of Finnish primary care.

**Figure 1:**
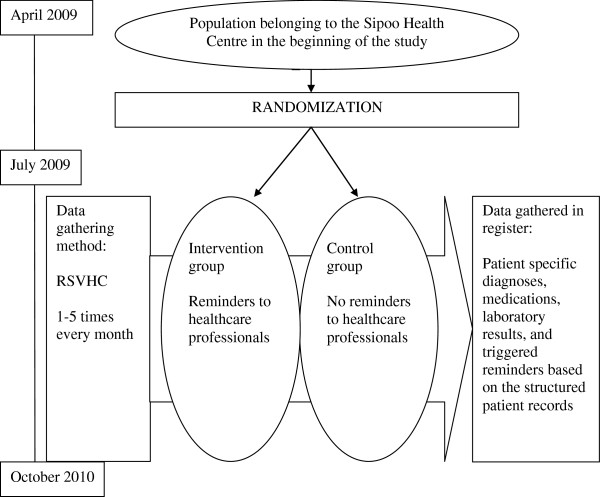
**Study design.** RSVHC is the Repeated Study Virtual Health Check where all decision support rules are run at once and the triggered reminders at the time point are registered; see text for explanation. Both the intervention and control groups were accrued as new individuals visited the health center (i.e. first contact date). Therefore, the starting and end points of follow-up are individual. Occupational healthcare was excluded. (See text and Figure 3 for further explanation).

We used a parallel randomized controlled trial design, with patient identification (ID) numbers in the EPR system as the unit of randomization. We made use of the Finnish Personal Identity Code (PIC), by which each individual can be specifically identified [25], to produce anonymized study IDs based on the PICs. All patients who were listed as undergoing occupational healthcare were excluded for legal reasons (Figure 1).

The study started in July 2009 and ended in October 2010.

We developed a unique method for population-based outcome data gathering from the EPR archive, the Repeated Study Virtual Health Check (RSVHC). During an RSVHC, the EPR archive sent to the EBMeDS service structured patient data (diagnoses, medications, and laboratory results) on the base study population (request), and the service generated all reminders triggered by these data and returned them (answer). The RSVHC was planned to be performed weekly at night. Actually, one to five RSVHCs were performed per month. The requests and answers of each RSVHC were stored automatically in a log file located in the EPR server, to be exported to the study register and analyzed at the end of the study period.

The reminders were written by the EBMeDS editorial team using medical evidence embedded in the sets of Duodecim guidelines and other sources, and linked with evidence-based decision support (DS) rules [26]. In April 2009, the 107 available DS rules were piloted in Sipoo. Subsequently, the chief medical officer of the health center decided which of the DS rules should be implemented. These totalled 96.

Here is an example of an implemented DS rule that conforms to if–then logic: ‘Metformin is the first choice oral hypoglycaemic agent in type 2 diabetes’ (DS rule 16).

The DS rule is implemented if the diagnosis in the EPR is type 2 diabetes. First, the rule checks whether the medication list on the EPR contains metformin. If it does not, the rule then checks for the plasma/serum creatinine value from the EPR laboratory results. If the glomerular filtration rate (GFR) is in the normal range, then reminder one, ‘Type 2 diabetes – start metformin*’* is shown on the screen. If the GFR is <60 ml/min, then reminder two, ‘Type 2 diabetes—start metformin, note GFR’ is shown. If the GFR is missing or out of date, then reminder three, ‘Type 2 diabetes—check renal function and start metformin’ is shown [26].

See Additional file 1 for more examples of DS rules and reminders targeting diabetes patients, a large patient group in primary care. HCPs can easily check background information and evidence behind each reminder by clicking the reminder and opening the references.

### Participants

This was a register-based study using the EPR data without any direct contact with patients.

The first step in the data collection comprised 52 RSVHCs carried out between July 2009 and October 2010 in the base population. At the end of the study, the population was 17,541 (total number of patient IDs in the study register). Data in the RSVHCs were structured patient-specific information (diagnoses, medications, laboratory results), and the triggered patient-specific reminders at each time point were stored in the study register.

In the second step, using the study ID, the earliest date was defined when the patient’s EPR had been opened during the study (start of individual follow-up). All EBMeDS procedures (requests and answers) which actually took place during the study were stored in monthly log files on the EPR server. One log file (April 2010) was missed because of technical problems. A baseline date was determined individually for each study patient as the date of the first opening of the EPR during the study period, and the patient-specific follow-up started from this date.

The third step involved linking the information from steps one and two. These final data comprised patient-specific information from the individual baseline date to all RSVHC points where the patient was followed up.

The swine flu epidemic, with the ensuing universal vaccination procedures, occurred between September 2009 and February 2010. We excluded from the monthly log files patients who received only the swine flu vaccination during a short visit (5 to 10 minutes) with a nurse. According to the nurses [27], nothing else was checked, including triggered reminders.

### Intervention

The intervention consisted of the patient-specific reminders being shown to the HCP on opening and using the EPR. A concrete example, the reminders for one diabetes patient, has been published previously [27]. Short versions of the triggered reminders, for example, ‘Type 2 diabetes—time for nephropathy screening’ , were shown automatically on screen. The full version of the reminders could be seen when the HCP hovered the cursor over the reminder, for example, ‘This patient has type 2 diabetes and no screening for microalbuminuria has been carried out during the last year. Annual screening for microalbuminuria is recommended in type 2 diabetes’. See Table 1 for examples of the reminders according to ICD-10 diagnosis groups. All study reminders are available in Additional files 2, 3 and 4.

**Table 1:** Examples of the EBMeDS reminders listed according to ICD-10 coding system

**Decision support ID**	**Decision support title**	**Reminder number**	**Reminder (short version)**
**Cardiovascular diseases (IX, Diseases of the circulatory system)**			
scr00457	Anticoagulants for atrial fibrillation	1	Atrial fibrillation—start warfarin?
		2	Atrial fibrillation—consider warfarin?
scr00578	Follow-up of patients with hypertension	1	Hypertension—time to check blood pressure?
		2	Elevated blood pressure in last measurement—time to check blood pressure?
**Ear diseases (VIII, Diseases of the ear and mastoid process)**			
scr00424	Avoiding decongestants and antihistamines in otitis media in children	1	Otitis media—avoid decongestants and antihistamines
**Endocrine and metabolic diseases (IV, Endocrine, nutritional and metabolic diseases)**			
scr00665	An abnormal potassium result	1	Serum potassium is dangerously out of range (@1)!
		2	Serum potassium is out of range (@1)
		3	Serum potassium is slightly out of range (@1)
**Genitourinary diseases (XIV Diseases of the genitourinary system)**			
scr00107	GFR below 55 ml/min	1	Decreased GFR—no diagnosis of renal failure
		2	Decreased GFR and no recent creatinine test—order new creatinine test?
**Haematological diseases (III, Diseases of the blood and blood-forming organs and certain disorders involving the immune mechanism)**			
scr00664	Low haemoglobin concentration in adults and adolescents	1	Decreased haemoglobin concentration—start investigations?
**Musculoskeletal diseases (XIII, Diseases of the musculoskeletal system and connective tissue)**			
scr00012	Prevention of osteoporosis in long-term use of glucocorticoids	1	Long-term glucocorticoids—add calcium and vitamin D?
		2	Long-term glucocorticoids—add a bisphosphonate?
**Neoplastic diseases (II, Neoplasms)**			
scr00094	Follow-up of high PSA concentration	1	High PSA—time to repeat the test?
		2	High PSA—time to repeat the test? Note 5-alpha reductase medication
**Nervous system diseases (VI, Diseases of the nervous system)**			
scr00425	SSRIs not indicated for headaches	1	Headache—SSRIs are not recommended
**Respiratory diseases (X, Diseases of the respiratory system)**			
scr00494	Inhaled corticosteroids instead of oral steroids for chronic asthma	1	Asthma treated with oral steroids—start inhaled steroids?
		2	Asthma treated with courses of oral steroids—start inhaled steroids?

(The decision support rule ID is included to assist interested readers to obtain more information at http://www.ebmeds.org).

The control group was treated according to normal practice, and the triggered patient-specific reminders were not shown to the HCP on screen. Instead, these were stored in the log files and exported to the study register. Usual care and the evidence for that were available to HCPs at all times during the trial, by active searching of, *e.g.*, guidelines.

There are four different EBMeDS decision support service functions: reminders, guideline links, a clinical virtual health check, and drug alerts (Table 2). Reminders and drug alerts are triggered automatically, but the guideline links and clinical virtual health check functions need active querying. Here, we focused only on the automatic reminder function. The interface of the integrated systems (EPR and EBMeDS) was designed by the EPR system vendor.

**Table 2:** The four EBMeDS decision support functions available for the healthcare professional

**Function**	**Description**
**Reminders** (See Table 1 and Additional file 2 for more detail)	These are patient-specific and *the short version* is shown automatically, *the long version* when the cursor hovers over the reminder. These are triggered on: opening of the patient record; or recording a new diagnosis; or prescribing new medication.
	The focus of the present paper.
Guideline links	These are shown in accordance with the patient’s diagnosis list and ICD-10 codes.
Virtual health check (VHC)	The healthcare professional can run a (clinical) VHC on a selected group of patients. Patient-specific reminders appear on the screen, which can be used *e.g.*, for planning the following day’s consultations.
Drug alerts	Reminders triggered on prescribing a medication.

**Figure 2:**
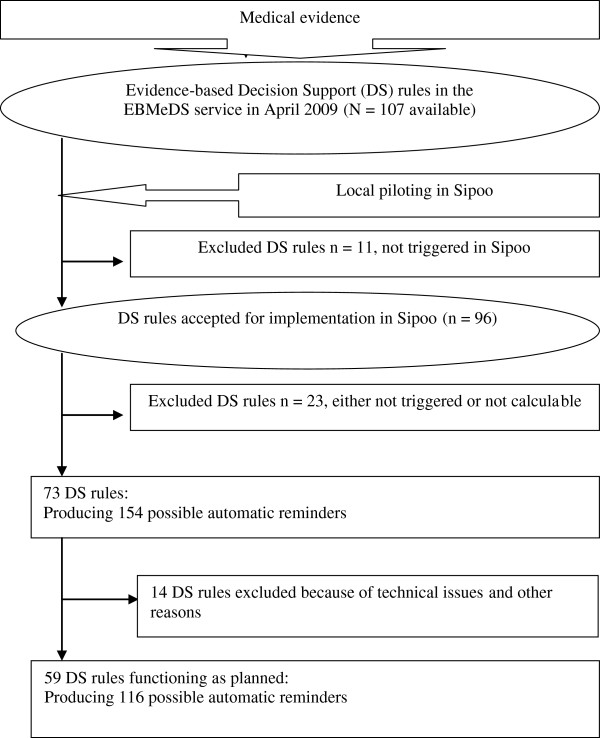
**The process of eliminating decision support rules from the analyses.** The process of elimination from the analyses of non-functioning decision support (DS) rules ending with the 59 rules that were used.

The number of reminders selected for the study was 154, based on 73 DS rules. After piloting, we had to exclude 23 of the original 96 DS rules (not triggered, n = 10, or not calculable, n = 13). In practice, 14 additional DS rules failed to function as planned (missing laboratory codes or unexpected changes in the EPR system after updates). After excluding a further 38 reminders based on these 14 DS rules, the analyzable final maximum number of different reminders was 116 (Figure 2).

### Outcomes

The primary composite outcome measure was the change in the numbers of all reminders triggered in the target population over 12 months of individual follow-up. As secondary outcome measures, we explored the changes also after three and six months of follow-up.

### Sample size

**Figure 3:**
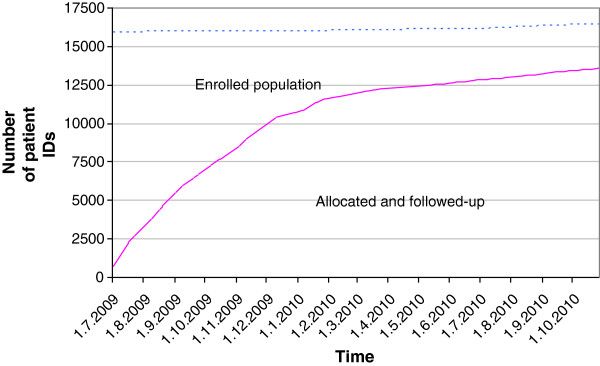
Accumulation of study participants from July 2009 to October 2010.

We planned to include all of the Sipoo patients’ EPRs in the study, and estimated that at least 50% of the population would contact the health center during one year, based on available data on visits to primary healthcare centers in Finland, and local statistics [28]. This translated into an approximation of 10,500 participants in the final study sample. The accumulation of the study participants (n = 13,588) is shown in Figure 3.

### Randomization

A single ratio procedure randomized the base population of the health center at the beginning of the study to an intervention and a control group of the same size without any other criteria. The procedure was done once per individual by a computer using a mathematical formula based on the PIC of each patient in the EPR system, and assigning each patient a unique study ID number. The forthcoming patients (for example, new inhabitants of Sipoo) were randomized according to the same procedure. The procedure was performed by a person outside the study group who also retained the key of the formula linking the PIC to the study ID number.

### Blinding

The randomization was masked from the patients, the HCPs, and the study group. However, when the HCP was shown the patient-specific reminders on screen, he or she knew that the patient belonged to the intervention group. The study group first opened randomization after the data collection period.

### Statistical methods

Baseline characteristics of the intervention and control group with ancillary analysis for triggered reminders were performed using means and standard deviations or frequencies and proportions (Table 3).

**Table 3:** Characteristics of the intervention and the control group in the three models

	**Model 1 with 12 months’ follow-up (indiv**^**1**^**_MO12)**		**Model 2 with 6 months’ follow-up (indiv**^**1**^**_MO6)**		**Model 3 with 3 months’ follow-up (indiv**^**1**^**_MO3)**	
	**Intervention**	**Control**	**Intervention**	**Control**	**Intervention**	**Control**
Number of patients						
	3,836	3,734	5,983	5,928	6,435	6,360
Age (mean, sd)						
	36.7 (27.8)	36.8 (27.6)	35.4 (26.0)	35.6 (25.8)	35.4 (25.7)	35.6 (25.6)
Gender (%)						
Female	1,997 (52.1)	1,960 (52.5)	3,068 (51.3)	3,020 (51.0)	3,305 (51.4)	3,254 (51.2)
Male	1,839 (47.9)	1,773 (47.5)	2,915 (48.7)	2,907 (49.0)	3,130 (48.6)	3,105 (48.8)
Number of diagnoses at baseline (%)						
0	1,253 (32.7)	1,220 (32.7)	2,335 (39.0)	2,325 (39.2)	2,576 (40.0)	2,568 (40.4)
1	913 (23.8)	862 (23.1)	1,385 (23.2)	1,344 (22.7)	1,467 (22.8)	1,433 (22.5)
2–3	995 (25.9)	985 (26.4)	1,402 (23.4)	1,392 (23.5)	1,490 (23.2)	1,460 (23.0)
4+	675 (17.6)	667 (17.8)	861 (14.4)	867 (14.6)	902 (14.0)	899 (14.1)
Number of diagnoses at the end of follow-up period (%)						
0	458 (11.9)	460 (12.3)	1,302 (21.8)	1,341 (22.6)	1,643 (25.5)	1,695 (26.7)
1	729 (19.0)	713 (19.1)	1,460 (24.4)	1,425 (24.0)	1,669 (25.9)	1,626 (25.6)
2–3	1,253 (32.7)	1,229 (32.9)	1,820 (30.4)	1,816 (30.6)	1,866 (29.0)	1,820 (28.6)
4+	1,396 (36.4)	1,332 (35.7)	1,401 (23.4)	1,346 (22.7)	1,257 (19.5)	1,219 (19.2)
Number of medications at baseline (%)						
0	1,877 (48.9)	1,809 (48.4)	3,464 (57.9)	3,388 (57.2)	3,797 (59.0)	3,702 (58.2)
1–5	1,363 (35.5)	1,322 (35.4)	1.877 (31.4)	1,870 (31.5)	1,977 (30.7)	1,976 (31.1)
6–9	368 (9.6)	372 (10.0)	403 (6.7)	425 (7.2)	413 (6.4)	434 (6.8)
10+	228 (6.0)	231 (6.2)	239 (4.0)	245 (4.1)	248 (3.9)	248 (3.9)
Number of triggered reminders at baseline (mean, sd)						
	0.30 (0.75)	0.31 (0.76)	0.23 (0.65)	0.23 (0.66)	0.23 (0.65)	0.23 (0.67)
Number of triggered reminders at the end of follow-up period (mean, sd)						
	0.45 (0.95)	0.45 (0.93)	0.27 (0.72)	0.28 (0.73)	0.24 (0.68)	0.25 (0.68)
Number of participants with no triggered reminder (%)						
	2,733 (71.2)	2,670 (71.5)	4,834 (80.8)	4,781 (80.7)	5,354 (83.2)	5,286 (83.1)

^1^Indiv = individual.

To investigate the effect of the intervention on patient care, the outcome variable was the number of triggered reminders in each RSVHC. Because the data were right-skewed and the variance was greater than the mean, the negative binomial model provided a better fit to the data and accounted for over-dispersion better than a Poisson regression model [29]. We used negative binomial regression to model the number of triggered reminders at 12, 6, and 3 months follow-up times (Table 4, models 1 to 3). The negative binomial model included a variable (group) to indicate the difference between groups at baseline and a variable (time) to indicate the changes in the number of triggered reminders over time. The difference in change in the number of triggered reminders across the intervention between the two groups was tested using an interaction term between group and time. The exponent of the coefficient of the interaction term is the incidence rate ratio (IRR), *i.e.*, an estimate of the relative difference in percentage change in the number of triggered reminders in the intervention group, compared with the control group. We also added to the models some potential confounding variables, such as age, gender, number of diagnoses, and number of medications.

**Table 4:** Incidence rate ratios (IRR) of the number of triggered reminders by negative binomial regression models using a generalized estimation equation

	**Unadjusted**			**Adjusted**^**e**^		
	**IRR (95% CI)**	**SE**^**d**^	**p-value**	**IRR (95% CI)**	**SE**^**d**^	**p-value**
Indiv_MO12^a^						
Group	1.002 (0.895 – 1.121)	0.057	0.98	1.004 (0.903 – 1.116)	0.054	0.94
Time	1.014 (1.001 – 1.023)	0.005	0.002	1.017 (1.008 – 1.026)	0.005	<0.001
Time^2^	1.002 (1.001 – 1.003)	0.0003	<0.001	1.002 (1.001 – 1.003)	0.0003	<0.001
Group × Time	1.001 (0.995 – 1.008)	0.003	0.73	1.002 (0.995 – 1.009)	0.003	0.56
Indiv_MO6^b^						
Group	1.011 (0.913 – 1.120)	0.053	0.84	1.008 (0.923 – 1.101)	0.045	0.86
Time	1.038 (1.030 – 1.046)	0.004	<0.001	1.044 (1.036 – 1.052)	0.004	<0.001
Group × Time	0.990 (0.980 – 1.001)	0.005	0.066	0.989 (0.978 – 0.9997)	0.005	0.044
Indiv_MO3^c^						
Group	0.990 (0.895 – 1.094)	0.051	0.84	1.013 (0.926 – 1.108)	0.046	0.77
Time	1.036 (1.024 – 1.050)	0.007	<0.001	1.046 (1.031 – 1.062)	0.008	<0.001
Group × Time	0.998 (0.980 – 1.017)	0.009	0.86	0.996 (0.975 – 1.018)	0.011	0.74

^a^Indiv_MO12 for the study patients with 12 months’ individual follow-up using exchangeable working correlation structure.

^b^Indiv_MO6 for the study patients with six months’ individual follow-up using exchangeable working correlation structure.

^c^Indiv_MO3 for the study patients with three months’ individual follow-up using the auto-regressive (AR1) working correlation structure.

^d^Semi-Robust Huber/White sandwich estimated standard errors.

^e^Adjusted for age, gender, number of diagnoses and number of medications.

To account for the within-participant correlation between repeated measures, we used Generalized Estimation Equations (GEE) by using the STATA software package (version 12.0 for Windows). Liang and Zeger proposed the GEE approach in 1986 to deal with impractical probability distribution in handling correlated responses [30]. We used different correlation structures (exchangeable, first-order autoregressive and unstructured) to account for the correlation within each unit. All models were evaluated in terms of how well they fitted the data using the quasi-likelihood under the independence model information criterion (QIC) for model selection [31]. The model with the lowest QIC was selected as the final model. Robust standard errors were used for all GEE-fitted models. IRRs were presented with 95% confidence intervals (95% CI) and p-values. We defined <0.05 risk of error as the significance p-value.

## Results

**Figure 4:**
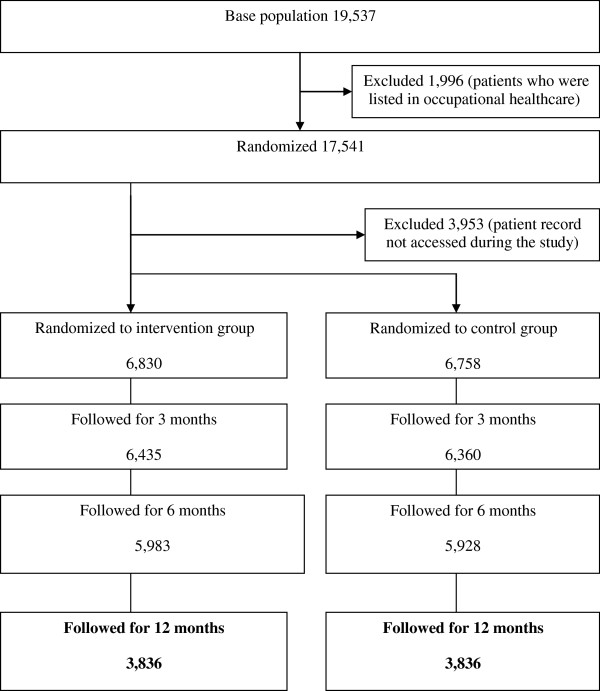
**Flow chart of study participants.** Follow-up time is individual. (Primary outcome measure shown in bold text).

In total, 17,541 potential participants were registered in the study on the basis of the 52 RSVHCs. Of these, 13,588 individuals’ EPRs were accessed by the HCPs during the study (Figure 4). The characteristics and descriptive statistics of the analyzed participants in different models (age, gender, number of diagnoses and triggered reminders at baseline and at the end of the follow-up period, number of medications at baseline, and number of participants with no triggered reminder) are presented in Table 3. The participants’ individual follow-up periods varied from 1 day to 480 days, which was decisive for inclusion in or exclusion from the GEE models’ analyses.

Three GEE models were made (Table 4). The primary outcome after 12 months, model indiv_MO12, included all participants with individual follow-up for 12 months (n = 7,570). At baseline, there were no differences between the intervention and control groups. The incidence rate for triggered reminders increased significantly (p = 0.002) over the follow-up period, and the intervention and control group behaved similarly. The result was congruent with confounding variables, such as age, gender, number of diagnoses, and number of medications (adjusted model).

The GEE model indiv_MO6 included all participants with at least six months of follow-up (n = 11,911). At baseline, the intervention and control groups did not differ. The incidence rate for triggered reminders increased significantly (p <0.001) during the follow-up period. The difference in development between the groups was not significant (p = 0.066) in the unadjusted model, but in the adjusted model there was a significant difference (p = 0.044), indicating that the number of reminders increased less in the intervention group than in the control group.

The GEE model indiv_MO3 included all participants with at least three months of follow-up (n = 12,795). At baseline, the intervention and control groups did not differ. During the follow-up period, the incidence rate for triggered reminders increased significantly (p <0.001). The intervention effect was not significant, indicating that the reminders were triggered similarly in both groups. The result was congruent with confounding variables in the adjusted model.

We did not detect any direct harm to the participants from the intervention during the trial. A conceivable harm to the HCPs originated from needless or incorrect reminders based on missing laboratory codes or unexpected changes in the EPR system after updates.

## Discussion

The two new data-gathering methods, the RSVHC and the monthly log file, functioned as planned. More than 70% of participants did not trigger reminders (Table 3), probably because most reminders were for chronic conditions and the proportion of elderly people is relatively small in the Sipoo community. Contrary to our expectations, the difference in the number of reminders after 12 months of follow-up (primary outcome measure) between the intervention and control group was not significant (Table 4), and the pattern was similar: increasing numbers of reminders in the intervention and the control group. We used the robust RCT method [32,33], and the most likely explanation for the results is that the recording of diagnostic codes improved markedly during the trial (Table 3). However, at six months individual follow-up time, the increase in the total number of reminders was significantly less in the intervention group than in the control group, when controlling for the confounding factors, such as age, gender, number of diagnoses, and medications.

### Limitations

There are a number of possible explanations for the results. First, the setting was one health center with 15 physicians making clinical decisions on diagnosis, medication, and laboratory tests. Because each physician took care of patients in the intervention group as well as in the control group, contamination was possible in so far as the physicians could have learned to treat the control group patients according to the reminders for the intervention group patients. This possible learning effect can be presumed, decreasing the trial effect. Therefore, the present results are a conservative estimate, and in future trials, a cluster randomization of several study sites or a randomization of HCPs would be preferable.

Second, the set of study reminders was chosen by the study group and may have addressed the HCPs’ needs insufficiently. Tailoring the guidance to HCPs’ needs has been indicated as a key issue for successful implementation [5,7]. Local HCPs, not only the Chief Medical Officer, may have to be involved for an adequate understanding of their needs as the starting point for developing and implementing reminders, as had been indicated previously [34]. Further, competence-based individual tailoring could be helpful.

Some of the most common and important reminders, including those warning of high LDL cholesterol, had to be excluded from the analysis because the codes for laboratory tests had changed over time (which the research group was not aware of), and only the old codes were interpreted by the rules, resulting in false reminders based on old (and not the most recent) test results. This may have resulted in mistrust of the reminders among the HCPs, and poor compliance with all reminders.

Many patients were seen by nurses rather than physicians, but the actions suggested by the reminders (like ordering tests or medications) could only be taken by physicians. The nurses may not always have consulted physicians after they saw the reminders, resulting in no action.

According to a meta-regression of 162 randomized trials [35], the odds of success of computer-based decision support are greater for systems that require HCPs to provide reasons when overriding advice than for systems that do not. The odds of success are also better for systems that provide advice concurrently to patients and HCPs. The intervention system possessed neither of these features.

There was a delay in implementing the EBMeDS service due to technical problems. This necessitated retraining the HCPs, which for practical reasons was delayed and took place in February 2010, eight months after the introduction of the service [24]. This delay from the introduction of the service to the training of the HCPs in its use may partly explain the results.

### Generalizability

The reliable performance of the data-gathering methods gives confidence that they can be applied successfully in future studies and combined with a more extensive use of decision support, for example the monitoring and auditing care of such large patient groups as those with type 2 diabetes.

We managed to randomize all patients of the health center as planned to two comparable study groups, indicating a high validity of the results [36-38]. However, the results may not be generalizable to other primary care environments, where the EBMeDS service could be more vigorously implemented. Another set of physicians in another health center could use the reminders much more or much less than the present 15 physicians, and therefore the results could be totally different. Also, the integration of the Mediatri EPR and the EBMeDS service was unique. That reminders were triggered by only below a third of the participants may be related to recording issues, or to the timing of the successive accessing of the EPR. Further exploration is warranted.

### Interpretation

The EBMeDS service, developed using the best available evidence [9,10], aimed to offer recommendations to HCPs’ workflow across several conditions in primary care practice. During the trial, patient-specific reminders were triggered systematically but had only limited effects on patient care (our secondary outcome measure after six months’ individual follow-up time). Our results reaffirm previous evidence [39-41] that implementation of computer-based decision support is problematic. HCPs seem to accept the service in principle [27,42], but in practice they may neglect using the reminders for many practical reasons.

The intervention itself is complex, as reminders have different purposes in accordance with decision support rules (Table 1 and Additional files). Some provide advice on diagnosis or medication or laboratory test decisions, for example ‘Atrial fibrillation—start warfarin?’ (DS rule 457), and some are reminders to follow patient measures, for example ‘Hypertension—time to check blood pressure?’ (DS rule 578). The expected time interval between consultations and changes in the patient record differs across the reminders: some changes take place quickly, even in one visit, for example, as the HCP records a new diagnosis or prescribes medication. By contrast, some changes need more time, at least until the next visit or laboratory test. In the latter case, the time interval between the triggering of the reminder and the measurement of the outcome was too long for reminders to show differences between the intervention and control groups. Groups of reminders and individual reminders should also be evaluated separately, probably after careful tailoring of the time to outcome, in order to determine the types of reminder that have an effect and when this should be measured.

Environmental issues included functional changes in the health center, such as turnover of staff, and the coincidence of the swine flu epidemic with the trial, which could have influenced the study. Moreover, the physicians decided that ICD-10 diagnosis classification would be used systematically from spring 2009. The use of the classification was made a mandatory function for recording patient data in each encounter. This might be a key reason for changes in data entry during the trial. However, in a randomized design both groups would have been affected similarly. The reason for a patient visit may not have had any bearing on the triggered reminders, resulting in HCPs ignoring them. In fact, our feasibility study [27] indicates that missing or outdated patient data on, for example, medication, resulted in needless or wrong reminders. These had to be checked in the EPR, which took time.

We can speculate on at least three specific issues. First, our hypothesis was based on an optimistic estimation of the potential consequences of triggered reminders. We assumed that if HCPs received patient-specific reminders they would act on them, and that this automatically would decrease the number of future reminders. We did not recognize that other factors in patient care—above all, changes in patient data recording—could have an opposite effect: for example, recording a new diagnosis or medication would trigger new reminders instead of decreasing the number of reminders triggered. This confirms previous findings [43,44].

Second, our choice of the primary outcome measure and its timing was based only on our understanding, without any actual evidence. Despite extensive research during the planning of the trial, we could not find a study to help us with this. Choosing the primary outcome measures is not easy [36].

Third, the analysis consisted of three models (Indiv_MO12, Indiv_MO6, Indiv_MO3) based on the different follow-up periods of the participants. In order to be followed up for 12 months and be included in the measurement of the primary outcome, a patient had to have a first contact by the end of October 2009. The starting date could be as late as April 2010 for inclusion in the six-month follow-up period and July for inclusion in the three-month period. Most notably, the groups of individuals with 3, 6, or 12 months’ follow-up differed in the numbers of diagnoses and medications at baseline (Table 3). The higher numbers of diagnoses, medications and triggered reminders in the 12-month group indicate more chronically ill patients in this group than in the other groups. The effects of the marked improvement in diagnosis coding during the study are difficult to assess, because the influence of reminders triggered for the intervention group may differ from that in the control group. Some of the reminders indicated to the HCP that diagnoses had not been coded. This may have resulted in more comprehensive coding of some diagnoses in the intervention group, which further may have resulted in more triggered reminders than in the control group, because many reminders could be triggered only if a specific diagnosis was present. Adjusting for the number of all diagnoses cannot fully remove this confounding factor.

## Conclusions

We did not find an intervention effect of the reminders on the primary outcome measure. However, a positive effect was seen in the secondary measure over a six-month follow-up period. This trial has to be considered a pilot, identifying key factors to be taken account when implementing and evaluating EBMeDS services or similar systems in the future. Patient information in the EPR system has to be accurately recorded for the reminders to trigger correctly. Appropriate functionality of the integrated system should be confirmed before the trial starts.

Presently, the integration of EBMeDS with any EPR system includes a thorough and systematic check of, for example, all existing laboratory code values. In integrated systems, all technical changes such as routine updating of the EPR system can influence the functioning of the decision support system.

## Competing interests

Authors TK, JR, MM, and PR declare that they have no competing interests. JK is Editor-in-chief of *Current Care Guidelines*, published by the Finnish Medical Society Duodecim, and a member of the editorial board for *EBMeDS*, Duodecim Medical Publications Ltd. IK is a salaried employee of Duodecim Medical Publications Ltd, the company that develops and licenses the EBMeDS decision support service. MK chairs the *Current Care Guidelines* board at Finnish Medical Society Duodecim. The authors declare that they have no competing interests.

## Authors’ contributions

All authors and other members of the EBMeDS study group (Jukkapekka Jousimaa, Helena Liira, Taina Mäntyranta and Peter Nyberg) were involved in conceiving the study and designing the trial. JR was responsible for data coding and analysis. TK led the writing process, supervised by MK, and all authors commented on sequential drafts and approved the final version of the manuscript.

## Supplementary Material

Additional file 1 **Examples of implemented decision support rules for diabetes and the reminders that may be triggered depending on the patient **[26]**.** Click here for file

Additional file 2 **All analysed reminders **[26]**.** The decision support rule ID is included to assist interested readers to obtain more information at http://www.ebmeds.org. Click here for file

Additional file 3 **Reminders that were excluded after local piloting **[26]**.** The decision support rule ID is included to assist interested readers to obtain more information at http://www.ebmeds.org. Click here for file

Additional file 4 **Decision support rules that were excluded from the analyses for technical and other reasons **[26]**.** The decision support rule ID is included to assist interested readers to obtain more information at http://www.ebmeds.org. Click here for file

## References

[B1] Haynes RB Devereaux PJ Guyatt GH Clinical expertise in the era of evidence-based medicine and patient choice ACP J Club 2002 136 2 A11 14 11874303

[B2] Burgers JS Grol R Klazinga NS Makela M Zaat J Towards evidence-based clinical practice: an international survey of 18 clinical guideline programs Int J Qual Healthcare 2003 15 1 31 45 10.1093/intqhc/15.1.31 12630799

[B3] Fixsen DL Naoom SF Blase KA Friedman RM Frances W Implementation research: a synthesis of the literature 2005 Tampa: University of South Florida, Louis de la Parte Florida Mental Health Institute, The National Implementation Research Network

[B4] Hojgaard L Forward look - implementation of medical research in clinical practice 2011 Strasbourg: European science foundation

[B5] Grol R Wensing M Eccles M Grol R, Wensing M, Eccles M Implementation of changes in practice Improving patient care: the implementation of change in clinical practice 2005 Edinburgh: Elsevier 6 14

[B6] Oxman AD Thomson MA Davis DA Haynes RB No magic bullets: a systematic review of 102 trials of interventions to improve professional practice CMAJ 1995 153 10 1423 1431 7585368 PMC1487455

[B7] Lugtenberg M Burgers JS Westert GP Effects of evidence-based clinical practice guidelines on quality of care: a systematic review Qual Saf Healthcare 2009 18 5 385 392 10.1136/qshc.2008.028043 19812102

[B8] Greenes RA Clinical decision support: the road ahead 2007 Boston: Academic

[B9] Garg AX Adhikari NK McDonald H Rosas-Arellano MP Devereaux PJ Beyene J Sam J Haynes RB Effects of computerized clinical decision support systems on practitioner performance and patient outcomes: a systematic review JAMA 2005 293 10 1223 1238 10.1001/jama.293.10.1223 15755945

[B10] Kawamoto K Houlihan CA Balas EA Lobach DF Improving clinical practice using clinical decision support systems: a systematic review of trials to identify features critical to success BMJ 2005 330 7494 765 10.1136/bmj.38398.500764.8F 15767266 PMC555881

[B11] Shortliffe EH Computer programs to support clinical decision making JAMA 1987 258 1 61 66 10.1001/jama.1987.03400010065029 3586293

[B12] Shojania KG Jennings A Mayhew A Ramsay CR Eccles MP Grimshaw J The effects of on-screen, point of care computer reminders on processes and outcomes of care Cochrane Database Syst Rev 2009 3 CD001096 10.1002/14651858.CD001096.pub2 PMC4171964 19588323

[B13] Bell LM Grundmeier R Localio R Zorc J Fiks AG Zhang X Stephens TB Swietlik M Guevara JP Electronic health record-based decision support to improve asthma care: a cluster-randomized trial Pediatrics 2010 125 4 e770 777 10.1542/peds.2009-1385 20231191

[B14] McCullough A Fisher M Goldstein AO Kramer KD Ripley-Moffitt C Smoking as a vital sign: prompts to ask and assess increase cessation counseling J Am Board Fam Med 2009 22 6 625 632 10.3122/jabfm.2009.06.080211 19897690

[B15] Padberg FT Jr Hauck K Mercer RG Lal BK Pappas PJ Screening for abdominal aortic aneurysm with electronic clinical reminders Am J Surg 2009 198 5 670 674 10.1016/j.amjsurg.2009.07.021 19887197

[B16] Schriefer SP Landis SE Turbow DJ Patch SC Effect of a computerized body mass index prompt on diagnosis and treatment of adult obesity Fam Med 2009 41 7 502 507 19582636

[B17] Balas EA Weingarten S Garb CT Blumenthal D Boren SA Brown GD Improving preventive care by prompting physicians Arch Intern Med 2000 160 3 301 308 10.1001/archinte.160.3.301 10668831

[B18] Car J Black A Anandan C Cresswell K Pagliari C McKinstry B Procter R Majeed A Sheikh A The impact of eHealth on the quality & safety of healthcare. A systematic overview & synthesis of the literature. Report for the NHS Connecting for Health Evaluation Programme. 2008 London: Imperial College London

[B19] Huckvale C Car J Akiyama M Jaafar S Khoja T Bin Khalid A Sheikh A Majeed A Information technology for patient safety Qual Saf Healthcare 2010 19 Suppl 2 i25 33 10.1136/qshc.2009.038497 20693213

[B20] Bryan C Boren SA The use and effectiveness of electronic clinical decision support tools in the ambulatory/primary care setting: a systematic review of the literature Inform Prim Care 2008 16 2 79 91 18713524 10.14236/jhi.v16i2.679

[B21] Gosling AS Westbrook JI Spencer R Nurses’ use of online clinical evidence J Adv Nurs 2004 47 2 201 211 10.1111/j.1365-2648.2004.03079.x 15196194

[B22] Gill JM Chen YX Glutting JJ Diamond JJ Lieberman MI Impact of decision support in electronic medical records on lipid management in primary care Popul Health Manag 2009 12 5 221 226 10.1089/pop.2009.0003 19848563

[B23] Sequist TD Gandhi TK Karson AS Fiskio JM Bugbee D Sperling M Cook EF Orav EJ Fairchild DG Bates DW A randomized trial of electronic clinical reminders to improve quality of care for diabetes and coronary artery disease J Am Med Inform Assoc 2005 12 4 431 437 10.1197/jamia.M1788 15802479 PMC1174888

[B24] Kortteisto T Komulainen J Kunnamo I Mäkelä M Kaila M Implementing clinical decision support for primary care professionals – the process Finn J eHealth eWelfare 2012 3 4 150 161

[B25] Electronic identity and certificates [http://www.vrk.fi/default.aspx?id=21]

[B26] EBMeDS clinical decision support [http://www.ebmeds.org/web/guest/home?lang=fi]

[B27] Kortteisto T Komulainen J Makela M Kunnamo I Kaila M Clinical decision support must be useful, functional is not enough: a qualitative study of computer-based clinical decision support in primary care BMC Health Serv Res 2012 12 349 10.1186/1472-6963-12-349 23039113 PMC3508894

[B28] SOTKAnet statistics and indicator bank 2005–2012 [http://uusi.sotkanet.fi/portal/page/portal/etusivu]

[B29] McCullagh P Nelder J Generalized linear models 1989 2 London: Chapman and Hall

[B30] Liang K-Y Zeger SL Longitudinal data analysis using generalized linear models Biometrika 1986 73 1 13 22 10.1093/biomet/73.1.13

[B31] Pan W Akaike’s information criterion in generalized estimating equations Biometrics 2001 57 1 120 125 10.1111/j.0006-341X.2001.00120.x 11252586

[B32] Campbell MJ Machin D Medical statistics: a commonsense approach 2002 3 Chichester: John Wiley & Sons, Ltd

[B33] Eccles M Grimshaw J Campbell M Ramsay C Research designs for studies evaluating the effectiveness of change and improvement strategies Qual Saf Healthcare 2003 12 1 47 52 10.1136/qhc.12.1.47 PMC1743658 12571345

[B34] Varonen H Kortteisto T Kaila M What may help or hinder the implementation of computerized decision support systems (CDSSs): a focus group study with physicians Fam Pract 2008 25 3 162 167 10.1093/fampra/cmn020 18504253

[B35] Roshanov PS Fernandes N Wilczynski JM Hemens BJ You JJ Handler SM Nieuwlaat R Souza NM Beyene J Van Spall HG Features of effective computerised clinical decision support systems: meta-regression of 162 randomised trials BMJ 2013 346 f657 10.1136/bmj.f657 23412440

[B36] Fransen GA van Marrewijk CJ Mujakovic S Muris JW Laheij RJ Numans ME de Wit NJ Samsom M Jansen JB Knottnerus JA Pragmatic trials in primary care. Methodological challenges and solutions demonstrated by the DIAMOND-study. BMC Med Res Methodol 2007 7 16 10.1186/1471-2288-7-16 17451599 PMC1865384

[B37] Juni P Altman DG Egger M Systematic reviews in healthcare: assessing the quality of controlled clinical trials BMJ 2001 323 7303 42 46 10.1136/bmj.323.7303.42 11440947 PMC1120670

[B38] Schulz KF Grimes DA Generation of allocation sequences in randomised trials: chance, not choice Lancet 2002 359 9305 515 519 10.1016/S0140-6736(02)07683-3 11853818

[B39] Black AD Car J Pagliari C Anandan C Cresswell K Bokun T McKinstry B Procter R Majeed A Sheikh A The impact of eHealth on the quality and safety of healthcare: a systematic overview PLoS Med 2011 8 1 e1000387 10.1371/journal.pmed.1000387 21267058 PMC3022523

[B40] Heselmans A Van de Velde S Donceel P Aertgeerts B Ramaekers D Effectiveness of electronic guideline-based implementation systems in ambulatory care settings - a systematic review Implement Sci 2009 4 82 10.1186/1748-5908-4-82 20042070 PMC2806389

[B41] Martens JD van der Weijden T Winkens RA Kester AD Geerts PJ Evers SM Severens JL Feasibility and acceptability of a computerised system with automated reminders for prescribing behaviour in primary care Int J Med Inform 2008 77 3 199 207 10.1016/j.ijmedinf.2007.05.013 17631412

[B42] Heselmans A Aertgeerts B Donceel P Geens S Van de Velde S Ramaekers D Family physicians’ perceptions and use of electronic clinical decision support during the first year of implementation J Med Syst 2012 36 6 3677 3684 10.1007/s10916-012-9841-3 22402980

[B43] Herzberg S Rahbar K Stegger L Schafers M Dugas M Concept and implementation of a computer-based reminder system to increase completeness in clinical documentation Int J Med Inform 2011 80 5 351 358 10.1016/j.ijmedinf.2011.02.004 21411365

[B44] Wright A Pang J Feblowitz JC Maloney FL Wilcox AR McLoughlin KS Ramelson H Schneider L Bates DW Improving completeness of electronic problem lists through clinical decision support: a randomized, controlled trial J Am Med Inform Assoc 2012 19 4 555 561 10.1136/amiajnl-2011-000521 22215056 PMC3384110

