# Supplementary material for: Patient-specific computer-based decision support in primary healthcare—a randomized trial
Source: Implement Sci. 2014 Jan 20;9:15. doi: 10.1186/1748-5908-9-15 (PMC3901002; doi:10.1186/1748-5908-9-15)
Supplement: Additional file 1 — Examples of implemented decision support rules for diabetes and the reminders that may be triggered depending on the patient [26]. [file 1748-5908-9-15-S1.pdf]

Additional file 1 – Examples of implemented decision support rules for diabetes and the reminders that may be triggered depending on the patient.

Decision support rule description	Reminder number	Reminder short version	Reminder long version
Metformin is the first choice oral hypoglycaemic agent in type 2 diabetes (scr00016)			
The script is launched if the diagnosis is type 2 diabetes. First, the script checks whether the drug list contains metformin. If it does not, the script checks for the plasma/serum creatinine value. If the GFR is in the normal range, reminder 1 is shown. If GFR < 60 ml/min, reminder 2 is shown. If GFR is missing or not fresh enough, reminder 3 is shown.	1	Type 2 diabetes - start metformin?	This patient has type 2 diabetes. Metformin is the drug of choice for better glycaemic control.
	2	Type 2 diabetes - start metformin? Note GFR.	This patient has type 2 diabetes. Metformin is the drug of choice for better glycaemic control. This patient's glomerular filtration rate, calculated with the MDRD formula (@1), is at a level where a lower than usual dose of metformin should be considered
	3	Type 2 diabetes - check renal function and start metformin?	This patient has type 2 diabetes. Metformin is the drug of choice for better glycaemic control. Consider checking renal function and starting metformin.
UKPDS Risk Engine to calculate cardiovascular and stroke risk in patients with type 2 diabetes (scr00129)			
The UKPDS calculators for cardiovascular and stroke risk are applied to the patient data. If the cardiovascular risk in 10 years exceeds 10% or the stroke risk exceeds 5%, a reminder is shown. If the smoking status or the presence of atrial fibrillation are not known, the reminders are shown if the risks exceed 10% or 5% by using the default settings "non-smoker" and "no atrial fibrillaton". If the 10-year risk of cardiovascular disease is above 10% reminder 1 (or 3) is shown. If the 10-year stroke risk is above 5% reminder 2 (or 4) is shown.	1	Type 2 diabetes - increased cardiovascular risk (UKPDS).	The risk of cardiovascular event is @1% in ten years according to the UKPDS risk engine.
	2	Type 2 diabetes - increased stroke risk (UKPDS).	This patient's ten-year risk of stroke is @1%, as calculated with the UKPDS Risk Engine.
	3	Type 2 diabetes, unknown smoking status - increased	This patient's ten-year risk of a cardiovascular event is @1%, as calculated with the UKPDS Risk Engine. Since information about this patient's smoking

Additional file 1 – Examples of implemented decision support rules for diabetes and the reminders that may be triggered depending on the patient.

		cardiovascular risk (UKPDS)	status was not found, the risk was calculated for a non-smoker. If the patient is a smoker, the ten-yea
	4	Type 2 diabetes, unknown smoking status - increased stroke risk (UKPDS)	This patient’s ten-year risk of stroke is @1%, as calculated with the UKPDS Risk Engine. Since information about this patient's smoking status was not found, the risk was calculated for a non-smoker. If the patient is a smoker, the ten-year risk is @2%.
Recall of patients with diabetes (scr00492)			
If none of the following laboratory tests has been taken during the last 13 months, a reminder on recalling the patient is shown: HbA1c, blood glucose, cholesterol.	1	Diabetes - time for the annual follow-up appointment?	This patient has diabetes. More than 13 months have passed since blood glucose and cholesterol were measured. Time for the annual follow-up appointment?
Screening for diabetic nephropathy in type 2 diabetes (scr00549)			
Albuminuria screening is recommended annually for patients with type 2 diabetes, if no result is available or the last result was negative.	1	Type 2 diabetes - time for nephropathy screening?	This patient has type 2 diabetes, and no screening for microalbuminuria has been carried out during the last year. Annual screening for microalbuminuria is recommended in type 2 diabetes.
Intensifying diabetes treatment in recently diagnosed type 2 diabetes (scr00564)			
If a patient with fresh (at most 2 years old) type 2 diabetes has an elevated HbA1c level (> 6,5 %) but no insulin treatment, the user is recommended to intensify hyperglycaemia treatment according to the flow-sheet described in the Finnish Current Care Guideline.	1	Type 2 diabetes and high HbA1c - intensify diabetes treatment?	This patient has type 2 diabetes and the HbA1c value is high (@1). Consider intensifying diabetes treatment.
