# Supplementary material for: Patient-specific computer-based decision support in primary healthcare—a randomized trial
Source: Implement Sci. 2014 Jan 20;9:15. doi: 10.1186/1748-5908-9-15 (PMC3901002; doi:10.1186/1748-5908-9-15)
Supplement: Additional file 2 — All analysed reminders [26]. The decision support rule ID is included to assist interested readers to obtain more information at http://www.ebmeds.org. [file 1748-5908-9-15-S2.pdf]

Additional file 2 – All analysed reminders

Decision support rule ID	Decision support title	Reminder number	Reminder short version
**Cardiovascular diseases (IX, Diseases of the circulatory system)**			
scr00001	Aspirin for nitrate users	1	Coronary heart disease? Start aspirin now?
		2	Coronary heart disease? Start aspirin? Note asthma.
scr00050	Checking for hyperthyroidism in recent onset atrial fibrillation	1	Atrial fibrillation - check thyroid function tests?
scr00109	Replacing atenolol with other antihypertensives	1	Atenolol as an antihypertensive agent? Replace with another drug?
scr00270	High BNP or proBNP; untreated heart failure	1	High BNP - untreated heart failure?
		2	Increased Nt-proBNP - untreated heart failure?
scr00271	Intensifying treatment of congestive heart failure because of high BNP or proBNP	1	Significantly high BNP - intensify heart failure treatment?
		2	Significantly high proBNP - intensify heart failure treatment?
scr00276	Follow-up of patients with congestive heart failure using spironolactone	1	This patient is taking spironolactone - check electrolytes?
		2	Spironolactone started - check electrolytes and renal function?
		3	Spironolactone and hyperkalaemia - stop spironolactone or reduce its dose?
		4	Spironolactone and hyperkalaemia – check potassium within 1 week.
		5	Spironolactone and impaired renal function - stop spironolactone?
		6	This patient is taking spironolactone - check renal function?
		7	Patient on spironolactone, and ACE/ARB started – check potassium and creatinine

Additional file 2 – All analysed reminders

	scr00280	Drug combinations not recommended for patients with congestive heart failure	1	Avoid the combination of an ACE inhibitor, angiotensin-receptor blocker and spironolactone
	scr00281	Harmful effects of NSAIDs in patients with congestive heart failure	1	Avoid NSAIDs in patients with heart failure
	scr00423	Pentoxifylline for venous leg ulcers	1	Chronic leg ulcer - consider pentoxifylline?
	scr00434	Indications for antihypertensive drug therapy based on total cardiovascular risk	1	High blood pressure - start antihypertensive drugs?
			2	High blood pressure and increased cardiovascular risk according to the SCORE system – start antihypertensive drugs after follow-up?
			3	Elevated blood pressure and increased cardiovascular risk according to the SCORE system – consider antihypertensive drugs?
			4	Elevated blood pressure and increased risk of cardiovascular disease according to the SCORE system. Check smoking status and start antihypertensive drugs?
			5	Elevated blood pressure and increased risk of cardiovascular disease according to the SCORE system. Check smoking status and start antihypertensive drugs after follow-up?
			6	Elevated blood pressure and increased risk of cardiovascular disease according to the SCORE system - check smoking status and consider antihypertensive drugs?
	scr00436	High SCORE risk in patients with unknown smoking status	1	High cardiovascular risk (SCORE) - check smoking status?
			2	Very high cardiovascular risk (SCORE) - check smoking status?

Additional file 2 – All analysed reminders

	scr00457	Anticoagulants for atrial fibrillation	1	Atrial fibrillation - start warfarin?
			2	Atrial fibrillation - consider warfarin?
	scr00462	Beta-blockers after myocardial infarction	1	Recent myocardial infarction - start a beta-blocker?
	scr00540	Aspirin plus dipyridamole for recurrent TIA	1	Recurrent TIA - add dipyridamole?
			2	Recurrent TIA - start aspirin + dipyridamole?
	scr00547	Smoking cessation for secondary prevention in atherosclerotic disease	1	Atherosclerotic disease - stop smoking?
			2	Atherosclerotic disease - attempt to stop smoking again?
	scr00578	Follow-up of patients with hypertension	1	Hypertension - time to check blood pressure?
			2	Elevated blood pressure in last measurement - time to check blood pressure?
	scr00588	The choice of NSAIDs in people with high cardiovascular risk	2	Increased cardiovascular risk - change diclofenac to paracetamol or naproxen?
			3	Increased cardiovascular risk - change high-dose ibuprofen to naproxen or non-NSAIDs?
			1	Increased cardiovascular risk - change the COX-2 inhibitor to paracetamol or naproxen?
			4	Increased cardiovascular risk - change the COX-2 inhibitor to paracetamol or naproxen?
			5	Increased cardiovascular risk - change diclofenac to paracetamol or naproxen?
			6	Increased cardiovascular risk - change high-dose ibuprofen to naproxen or non-NSAIDs?
	scr00610	Avoiding the combination of an anticoagulant and antiplatelet agent in peripheral arterial disease	1	Peripheral arterial disease - avoid the combination of an anticoagulant and antiplatelet agent?

Additional file 2 – All analysed reminders

		2	Peripheral arterial disease - avoid the combination of an anticoagulant and antiplatelet agent? Note: Recent coronary stenting
scr00675	Indications for antihypertensive drug therapy in patients with cardiovascular disease, renal disease or diabetes	1	Hypertension and a disease which may increase cardiovascular risk - treat hypertension?
**Congenital abnormalities (XVII, Congenital malformations, deformations and chromosomal abnormalities/Chromosomal abnormalities)**			
scr00046	TSH screening in Down syndrome	1	Down syndrome - check TSH?
		2	Down syndrome and high TSH - hypothyroidism?
		3	Down syndrome and low TSH - hyperthyroidism?
**Ear diseases (VIII, Diseases of the ear and mastoid process)**			
scr00424	Avoiding decongestants and antihistamines in otitis media in children	1	Otitis media - avoid decongestants and antihistamines
**Endocrine and metabolic diseases(IV, Endocrine, nutritional and metabolic diseases)**			
scr00016	Metformin is the first choice oral hypoglycaemic agent in type 2 diabetes	1	Type 2 diabetes - start metformin?
		2	Type 2 diabetes - start metformin? Note GFR.
		3	Type 2 diabetes - check renal function and start metformin?
scr00019	ACE inhibitor or sartan for diabetic patients with albuminuria	1	Diabetes and microalbuminuria - start ACE inhibitor or sartan?
		2	Diabetes and albuminuria - start ACE inhibitor or sartan?
scr00025	Glimepiride warning in renal insufficiency	1	Glimepiride treatment - impaired renal function noted?
		2	Discontinue glimepiride due to impaired renal function?

Additional file 2 – All analysed reminders

	scr00026	Glipizide warning in renal insufficiency	1	Glipizide treatment - impaired renal function noted?
			2	Discontinue glipizide due to impaired renal function?
	scr00030	Interpreting abnormal thyroid function test results	1	Abnormal thyroid function test results - untreated hyperthyroidism?
			2	Abnormal thyroid function test results - untreated hypothyroidism?
			3	Slightly increased TSH - repeat the test
			4	Abnormal thyroid function test results - untreated hypothyroidism?
			5	Abnormal thyroid function tests - untreated hyperthyroidism?
	scr00129	UKPDS Risk Engine to calculate cardiovascular and stroke risk in patients with type 2 diabetes	1	Type 2 diabetes - increased cardiovascular risk (UKPDS).
			2	Type 2 diabetes - increased stroke risk (UKPDS).
			3	Type 2 diabetes, unknown smoking status - increased cardiovascular risk (UKPDS)
			4	Type 2 diabetes, unknown smoking status - increased stroke risk (UKPDS)
	scr00492	Recall of patients with diabetes	1	Diabetes - time for the annual follow-up appointment?
	scr00508	Hypertension and hypokalaemia	1	A thiazide and hypokalaemia - change to a potassium-sparing diuretic?
			2	Hypokalaemia whilst using potassium-sparing diuretics – consult a specialist physician?
			3	Hypertension and hypokalaemia - hyperaldosteronism ruled out?
			4	Hypertension and significant hypokalaemia - rule out hyperaldosteronism and treat hypokalaemia
			5	Hypertension and hypokalaemia in the last measurement - re-check potassium?

	scr00549	Screening for diabetic nephropathy in type 2 diabetes	1	Type 2 diabetes - time for nephropathy screening?
	scr00550	Nephropathy screening in type 1 diabetes	1	Type 1 diabetes - time for nephropathy screening?
	scr00564	Intensifying diabetes treatment in recently diagnosed type 2 diabetes	1	Type 2 diabetes and high HbA1c - intensify diabetes treatment?
	scr00581	ACE inhibitors or angiotensin-receptor blockers for patients with diabetes and hypertension but no microalbuminuria	1	Diabetes and hypertension - start an ACE inhibitor or angiotensin-receptor blocker to prevent microalbuminuria?
	scr00663	Poorly controlled hypothyroidism	1	Hypothyroidism - too high thyroxine dose?
			2	Hypothyroidism - too low thyroxine dose?
	scr00665	An abnormal potassium result	1	Serum potassium is dangerously out of range (@1)!
			2	Serum potassium is out of range (@1)
			3	Serum potassium is slightly out of range (@1)
**Gastrointestinal diseases (XI, Diseases of the digestive system)**				
	scr00513	PPIs for maintenance therapy of reflux oesophagitis	1	If gastroesophageal reflux disease becomes symptomatic, consider proton pump inhibitors (PPIs)?
**Genitourinary diseases (XIV Diseases of the genitourinary system)**				
	scr00107	GFR below 55 ml/min	2	Decreased GFR and not recent creatinine test - order new creatinine test?
			1	Decreased GFR - no diagnosis of renal failure
	scr00428	Managing male sexual dysfunction associated with SSRIs	1	Erectile dysfunction - start a PDE-5 inhibitor?
	scr00444	ACE inhibitors for hypertension in patients with non-diabetic renal disease	1	Renal hypertension? Consider an ACE inhibitor?

Additional file 2 – All analysed reminders

scr00482	Local oestrogen for vaginal atrophy in postmenopausal women	1	Vaginal atrophy and possible menopause - start local oestrogen?
		2	Vaginal atrophy after oophorectomy - start local oestrogen?
scr00512	NSAIDs contributing to acute renal failure	1	Low GFR - consider the role of NSAIDs?
scr00538	Limiting the duration of hormone replacement therapy	1	Time to discontinue HRT?
**Haematological diseases (III, Diseases of the blood and blood-forming organs and certain disorders involving the immune mechanism)**			
scr00554	Low-dose aspirin for patients with polycythaemia vera	1	Polycythaemia vera - start low-dose aspirin as prophylaxis against thrombosis?
		2	Polycythaemia vera - start low-dose aspirin as prophylaxis against thrombosis? Note: previous peptic ulcer.
scr00664	Low haemoglobin concentration in adults and adolescents	1	Decreased haemoglobin concentration - start investigations?
**Infectious diseases (I, Certain infectious and paracitic diseases)**			
scr00677	Follow-up and contact tracing in sexually transmitted chlamydia infections	1	New case of chlamydia - retest after treatment and facilitate testing of partners!
		2	A recently diagnosed chlamydia - verify that the treatment was successful!
**Mental and behavioural disorders (V)**			
scr00340	WBC monitoring during clozapine treatment	1	Clozapine treatment - check WBC count at least twice weekly
		2	Clozapine treatment and decreased WBC count - discontinue clozapine, check WBC count daily
		3	Clozapine treatment - check WBC count?
scr00470	Continuing antipsychotics in patients with schizophrenia	1	Recent acute episode of schizophrenia - continue antipsychotic treatment?
scr00516	SSRIs for post-traumatic stress disorder	1	Post-traumatic stress disorder (PTSD) - consider using SSRIs?

Additional file 2 – All analysed reminders

**Musculoskeletal diseases (XIII, Diseases of the musculoskeletal system and connective tissue)**			
scr00012	Prevention of osteoporosis in long-term use of glucocorticoids	1	Long-term glucocorticoids - add calcium and vitamin D?
		2	Long-term glucocorticoids – add a bisphosphonate?
scr00035	Stopping bisphosphonates after 5 years of treatment	1	Time to stop bisphosphonate treatment?
scr00676	Follow-up tests for antirheumatic drugs	1	Antirheumatic drug therapy - follow-up tests due?
**Neoplastic diseases (II, Neoplasms)**			
scr00094	Follow-up of high PSA concentration	1	High PSA - time to repeat the test?
		2	High PSA - time to repeat the test? Note 5-alpha reductase medication
scr00464	Bisphosphonates for myeloma	1	This patient has myeloma - start bisphosphonate treatment?
**Nervous system diseases (VI, Diseases of the nervous system)**			
scr00425	SSRIs not indicated for headaches	1	Headache - SSRIs are not recommended
**Respiratory diseases (X, Diseases of the respiratory system)**			
scr00422	Cardioselective beta-blockers for patients with asthma/COPD	1	Obstructive pulmonary disease - change to a selective beta-blocker?
scr00450	Inhaled corticosteroids preferred to anti-leukotrienes in the treatment of asthma	1	Consider inhaled corticosteroids?
scr00494	Inhaled corticosteroids instead of oral steroids for chronic asthma	1	Asthma treated with oral steroids - start inhaled steroids?
		2	Asthma treated with courses of oral steroids - start inhaled steroids?
scr00534	Drugs for preventing frequent exacerbations of COPD	1	Frequent COPD exacerbations - add tiotropium?
		2	Frequent COPD exacerbations - add a mucolytics?

Additional file 2 – All analysed reminders

			3	Frequent COPD exacerbations - add an inhaled steroids?
	scr00539	Intranasal steroids for nasal polyposis	1	Nasal polyposis - start intranasal steroids?
