# Supplementary material for: Patient-specific computer-based decision support in primary healthcare—a randomized trial
Source: Implement Sci. 2014 Jan 20;9:15. doi: 10.1186/1748-5908-9-15 (PMC3901002; doi:10.1186/1748-5908-9-15)
Supplement: Additional file 3 — Reminders that were excluded after local piloting [26]. The decision support rule ID is included to assist interested readers to obtain more information at http://www.ebmeds.org. [file 1748-5908-9-15-S3.pdf]

Additional file 3 – Reminders that were excluded after local piloting.

Decision support rule ID	Decision support title	Reminder number	Reminder short version	Reason for exclusion
**Cardiovascular diseases (IX, Diseases of the circulatory system)**				
scr00279	Initial dose of angiotensin-receptor blockers in patients with congestive heart failure	1	Losartan treatment about to start - too high initial dose?	not triggered in population
		2	Candesartan treatment about to start - too high initial dose?	
		3	Valsartan treatment about to start - too high initial dose?	
scr00426	Aspirin after endovascular treatment or bypass surgery to the lower limbs	1	This patient has undergone endovascular treatment - aspirin is indicated.	not triggered in population
scr00437	Need to intensify dyslipidaemia treatment in patients with established cardiovascular disease	1	Cardiovascular disease and high LDL-cholesterol - intensify dyslipidaemia treatment?	not calculable
		2	Cardiovascular disease and LDL-cholesterol slightly above target	
		3	Diabetes, microalbuminuria and high LDL-cholesterol - intensify dyslipidaemia treatment?	
		4	Diabetes and LDL-cholesterol slightly above target	
scr00577	Drug therapy for severe pulmonary hypertension	1	Severe pulmonary hypertension - intensify treatment?	not triggered in population
Endocrine and metabolic diseases(IV, Endocrine, nutritional and metabolic diseases)				
scr00020	Routine procedures when starting a glitazone	1	Glitazone treatment about to start - contraindicated due to heart failure?	not calculable

Additional file 3 – Reminders that were excluded after local piloting.

Decision support rule ID	Decision Support title	Reminder number	Reminder short version	Reason for exclusion
		2	Glitazone treatment about to start - contraindicated due to insulin treatment?	
		3	Glitazone treatment about to start - information given about the risk of anaemia?	
		4	Glitazone treatment about to start – possible interactions noted?	
		5	Glitazone treatment about to start - weight monitoring arranged?	
scr00086	Diabetes risk test for patients with BMI above 30	1	Overweight - diabetes risk test indicated	not calculable
scr00090	Diagnosing T3 hyperthyroidism	1	Low TSH but normal T4 - check T3?	not calculable
scr00265	Laboratory results in patients with Type 1 diabetes	1	Type 1 diabetes with poor glycaemic control	not calculable
		2	Type 1 diabetes with suboptimal glycaemic control	
		3	Type 1 diabetes and high LDL-cholesterol	
		4	Type 1 diabetes and raised LDL-cholesterol	
		5	Type 1 diabetes and renal failure	
		6	Type 1 diabetes and proteinuria	
		7	Type 1 diabetes and microalbuminuria	
Gastrointestinal diseases (XI, Diseases of the digestive system)				
scr00509	Helicobacter eradication in patients with a bleeding ulcer	1	Bleeding peptic ulcer - Helicobacter pylori successfully eradicated?	not triggered in population
Genitourinary diseases (XIV Diseases of the genitourinary system)				

Additional file 3 – Reminders that were excluded after local piloting.

Decision support rule ID	Decision support title	Reminder number	Reminder short version	Reason for exclusion
scr00070	Anaemia in patients with menorrhagia	1	Menorrhagia and severe anaemia - consult a specialist?	not calculable
		2	Menorrhagia and moderate anaemia - start investigations into the cause of anaemia?	
		3	Menorrhagia - check haemoglobin?	
scr00006	Polycystic ovary syndrome (PCOS) and metabolic risks	1	PCOS - give information about risks related to metabolic syndrome.	not triggered in population
		2	PCOS - check blood glucose?	
		3	PCOS - check lipid profile?	
		4	PCOS and the patient is overweight - suggest weight reduction?	
scr00535	SSRIs in premenstrual syndrome	1	Recurrent premenstrual syndrome - try SSRIs?	not triggered in population
scr00599	Phosphodiesterase inhibitors for erectile dysfunction in patients with diabetes mellitus	1	Erectile dysfunction and diabetes - try a phosphodiesterase inhibitor?	not triggered in population
Haematological diseases (III, Diseases of the blood and blood-forming organs and certain disorders involving the immune mechanism)				
scr00091	Diagnosing polycythaemia vera	1	High haemoglobin with pancytosis - polycythaemia vera?	not calculable
Infectious diseases (I, Certain infectious and paracitic diseases)				
scr00528	Metronidazole in preference to vancomycin as the first choice in Clostridium difficile diarrhoea	1	Clostridium difficile diarrhoea - consider using metronidazole instead of vancomycin?	not triggered in population
Mental and behavioural disorders (V)				
scr00043	High MCV in men suggesting alcohol abuse	1	Increased MCV without anaemia - alcohol abuse?	not calculable

Additional file 3 – Reminders that were excluded after local piloting.

Decision support rule ID	Decision support title	Reminder number	Reminder short version	Reason for exclusion
scr00103	Depression associated with other diseases, medications or addictive substances	1	Newly diagnosed depression - possible aetiological factors?	not calculable
		2	Newly diagnosed depression - possible aetiological factors?	
		3	Newly diagnosed depression - possible aetiological factors?	
scr00124	Laboratory tests before antipsychotic treatment	1	Antipsychotic medication about to start - test results missing?	not calculable
scr00125	Laboratory tests before SSRI treatment	1	SSRI medication about to start - check full blood count (including platelets)?	not calculable
		2	SSRI medication about to start in a patient with diabetes - check fasting glucose?	
		3	SSRI medication about to start in a patient taking warfarin - check INR?	
scr00126	Laboratory tests before tricyclic antidepressant treatment	1	Tricyclic antidepressant treatment about to start - check appropriate laboratory tests?	not triggered in population
scr00127	Laboratory tests before duloxetine, milnacipran, venlafaxine and trazodone treatment	1	Antidepressive medication about to start - check full blood count (including platelets)?	not calculable
		2	Antidepressive medication about to start – check ECG if the patient has cardiac symptoms.	
		3	Antidepressive medication about to start - check INR?	
Musculoskeletal diseases (XIII, Diseases of the musculoskeletal system and connective tissue)				

Additional file 3 – Reminders that were excluded after local piloting.

Decision support rule ID	Decision support title	Reminder number	Reminder short version	Reason for exclusion
scr00604	Topical NSAIDs for lateral epicondylitis	1	Lateral epicondylitis - try topical NSAIDs?	not triggered in population
Respiratory diseases (X, Diseases of the respiratory system)				
scr00592	Antibiotics for COPD exacerbation	1	COPD exacerbation - consider antibiotics?	not calculable
