# Supplementary material for: Patient-specific computer-based decision support in primary healthcare—a randomized trial
Source: Implement Sci. 2014 Jan 20;9:15. doi: 10.1186/1748-5908-9-15 (PMC3901002; doi:10.1186/1748-5908-9-15)
Supplement: Additional file 4 — Decision support rules that were excluded from the analyses for technical and other reasons [26]. The decision support rule ID is included to assist interested readers to obtain more information at http://www.ebmeds.org. [file 1748-5908-9-15-S4.pdf]

Additional file 4 – Decision support rules that were excluded from the analyses for technical and other reasons

Decison support rule ID	Decision support title	Reminder number	Reminder short version
**Cardiovascular diseases (IX, Diseases of the circulatory system)**			
scr00435	Indications for lipid-lowering drugs based on total cardiovascular risk	1	High cardiovascular risk (SCORE) - add a statin?
		2	Increased LDL-cholesterol - lifestyle advice to reduce lipid values?
		3	High cardiovascular risk (SCORE) - add a statin?
		4	Increased LDL-cholesterol - lifestyle advice to reduce cholesterol?
scr00447	Follow-up of patients who are taking amiodarone	1	Amiodarone treatment about to start - check thyroid function?
		2	Amiodarone treatment about to start - check liver function?
		3	Amiodarone treatment about to start - check spirometry?
		4	Amiodarone treatment about to start - check chest X-ray?
		5	Amiodarone treatment - monitor thyroid function.
		6	Amiodarone treatment - monitor liver function.
		7	Amiodarone treatment - regular ECGs?
		8	Amiodarone treatment - regular chest X-rays?
scr00500	Glucose tests for patients with hypertension, dyslipidaemia or cardiovascular disease	1	Hypertension and no recent glucose values - check blood glucose?
		2	Hyperlipidaemia and no recent glucose values - check blood glucose?
		3	Atherosclerotic disease and no recent glucose values - check blood glucose?
**Endocrine and metabolic diseases(IV, Endocrine, nutritional and metabolic diseases)**			
scr00022	Intensifying dyslipidaemia treatment in patients with type 2 diabetes	1	Type 2 diabetes – adequate measures taken to manage dyslipidaemia?
		2	Type 2 diabetes and arterial disease – adequate measures taken to manage dyslipidaemia?

Additional file 4 – Decision support rules that were excluded from the analyses for technical and other reasons

Decison support rule ID	Decision support title	Reminder number	Reminder short version
scr00108	Aspirin for patients with type 2 diabetes	1	Type 2 diabetes - start aspirin?
		2	Type 2 diabetes - start aspirin? Note asthma.
scr00110	Increased fasting blood glucose	1	Increased blood glucose - diabetes?
		2	Significantly increased blood glucose - diabetes?
scr00113	OGTT follow-up after gestational diabetes	1	History of gestational diabetes – oral glucose tolerance test indicated?
scr00269	LDL-cholesterol concentration in patients with Type 2 diabetes	1	Type 2 diabetes and high LDL-cholesterol
		2	Type 2 diabetes and raised LDL-cholesterol
		3	Type 2 diabetes and arterial disease - note raised LDL-cholesterol
		4	Type 2 diabetes - no results for LDL-cholesterol available
scr00490	Glucose and lipid tests for patients with BMI above 32	1	High BMI – check blood glucose?
		2	Overweight? Check blood glucose?
		3	High BMI - lipid profile due?
		4	Overweight - lipid profile due?

Gastrointestinal diseases (XI, Diseases of the digestive system)

scr00128	Transglutaminase antibodies instead of gliadin antibodies both for screening and follow-up of coeliac disease	1	Gliadin antibodies checked - check also transglutaminase antibodies?

Genitourinary diseases (XIV Diseases of the genitourinary system)

scr00005	Selection of diuretics for people with renal insufficiency	1	Increased creatinine - change thiazide to furosemide?
		2	Thiazide treatment - check creatinine?
scr00085	Calcium x phosphate product in patients with renal failure	1	Renal failure and an increased calcium x phosphate product - treatment needed?

Musculoskeletal diseases (XIII, Diseases of the musculoskeletal system and connective tissue)

Additional file 4 – Decision support rules that were excluded from the analyses for technical and other reasons

Decison support rule ID	Decision support title	Reminder number	Reminder short version
scr00083	Folate for patients taking methotrexate	1	Methotrexate treatment - add folate?
**Pregnancy (XV, Pregnancy, childbirth and puerperium)**			
scr00453	Antibiotics for asymptomatic bacteriuria in pregnancy	1	This patient is pregnant - prescribe antibiotics for bacteriuria?
